# Demographic Disparities in Late-Stage Diagnosis of Breast and Colorectal Cancers Across the USA

**DOI:** 10.1007/s40615-016-0219-y

**Published:** 2016-04-12

**Authors:** Lee R. Mobley, Tzy-Mey Kuo

**Affiliations:** 1 School of Public Health and Andrew Young School of Policy Studies, Georgia State University, Atlanta, GA USA; 2 Lineberger Cancer Center, University of North Carolina at Chapel Hill, Chapel Hill, NC USA

**Keywords:** Geographic disparities, Cancer disparities, Population health, Multilevel modeling

## Abstract

**Background:**

We determined whether there were disparities in the likelihood of being diagnosed at a late stage for breast cancer (BC) or colorectal cancer (CRC) in each of 40 states, using the recently available US Cancer Statistics (USCS) database.

**Methods:**

We extracted 981,457 BC cases and 558,568 CRC cases diagnosed in 2004–2009. Separate multilevel regressions were run for each state and each cancer type. Models included person and area-level covariates and were identically specified across states. The disparities foci were race or ethnicity (white, African-American, Hispanic, Asian, all other), gender, and age (<40, 40–49, 50–64, 65–74, and 75+). Using whites, males, and the oldest age group as reference groups, we noted the statistically significant disparities coefficients (*p* value ≤0.05) and translated the findings via a set of maps of states in the USA.

**Results:**

National disparity estimates were not consistent with disparities identified in the states. Some states had estimates consistent with the national average, while others did not. Patterns of disparities across states were different for each covariate and mapped separately.

**Conclusion:**

National disparity estimates may mask what is true at the more local, state level because national estimates can confound the effects of race with place. Cancer control efforts are local and require locally relevant information to assess needs. Findings from the period 2004–2009 establish valuable benchmarks against which to assess changes following national health reform implemented in 2010. The USCS database is a valuable new resource that will facilitate future disparities research.

## Introduction

Cancer is the second most common cause of death in the USA, exceeded only by heart disease, and accounts for nearly one of every four deaths [6, 30]. In the USA, breast cancer (BC) is the most common cancer in women and colorectal cancer (CRC) is the third most common cancer in both men and women [6, 17]. There are disparities across population subgroups in the incidence, mortality, and the likelihood of cancer being diagnosed at a late stage—which is associated with greatly increased risk of mortality from cancer.

Incidence and death rates for CRC are about 35–40 % higher in men than in women and are highest among African-American men and women. African-American incidence rates are about 20 % higher, and mortality rates are about 45 % higher than those in whites. Hispanics, Asians, and Native Americans have lower incidence and mortality rates than whites or African-Americans. However, Native Americans have disproportionately high mortality as compared to the low incidence rates. Incidence and death rates for CRC increase with age; about 90 % of new cases and 94 % of deaths occur in individuals 50 and older. Geographic differences in incidence and mortality rates for CRC are considerable [5].

By comparison, BC incidence rates are highest among non-Hispanic white women, followed by African-American women, with lowest rates among Hispanic women. Asians, Native Americans, and Hispanics have lower mortality rates than non-Hispanic whites or African-Americans, with lowest rates for Asian women. Despite higher incidence rates among non-Hispanic whites, BC death rates are generally lower as compared to African-American women. BC incidence and death rates generally increase with age; 95 % of new cases and 97 % of deaths occur in women 40 and older. Similar to CRC, geographic differences in incidence and mortality rates for BC are considerable [4].

To understand why higher incidence is not always accompanied by higher mortality, it is important to examine differences in the stage at which cancer is diagnosed. Both BC and CRC screening rates are lower than optimal, resulting in later-staged cancers at diagnosis [3, 6]. The high proportions of late-stage diagnoses remain a public health concern, as it results in higher morbidity and mortality than would obtain with optimal cancer screening utilization [2, 3, 14, 29, 31]. The 5-year survival rate for persons who received a diagnosis of localized stage of CRC is 91 %, compared with 70 % for regional stage and 11 % for distant stage. Similarly, the 5-year survival rate for women who receive a diagnosis of localized stage of BC is 98 %, compared with 84 % for regional stage and 23 % for distant stage [16].

Henley et al. [14]) used comprehensive national cancer data 2004–2006 and examined disparities in late-stage diagnosis of cancer by age and by race or ethnicity for people aged 50 or older. Roughly a third of new BC cases and roughly half of new CRC cases were diagnosed at a late stage. The rates of late-stage BC and CRC incidence were calculated for each age group, gender, or race or ethnicity from pooled national data. Asians and Pacific Islanders, American Indians and Alaska Natives, and Hispanics had consistently lower late-stage rates than whites or African-Americans, for both cancer types. The rates of late-stage CRC incidence increased with age and were highest among African-American men and women. Late-stage BC incidence rates increased with age only through age 79 and were highest among the age 60–79 group and African-American women.

National Cancer Institute statistics online show that late-stage diagnosis rates are higher among younger populations than among older populations for both BC and CRC [24]. One recent study used the same national cancer data as Henley et al. [14]) but over a longer time horizon (2004–2009) and included cancer patients younger than age 50 to examine predictors of late-stage disparities for BC and CRC. Using multilevel logistic regression of national data pooled across states, accounting for a variety of personal and place-specific factors, they found that younger people (under age 65) had much higher likelihood of late-stage diagnosis of BC or CRC than older people; African-Americans had higher rates of late-stage diagnosis than whites; and other races or ethnicities (combined) had lower rates of late-stage diagnosis than whites [21].

Several additional studies have used regression modeling to examine various predictors of stage at diagnosis for BC and CRC, using smaller data sets. Studies have examined low SES, marital status, race or ethnicity, distance to closest provider, managed care penetration, area screening rates, residence in a racially segregated community, area poverty or deprivation, lack of personal insurance, or percent uninsured in the area, and type of personal health insurance as predictors [7, 12, 15, 18, 19, 33]. Area poverty or deprivation, lack of personal insurance, or percent uninsured in the area predicted higher likelihood of late-stage BC diagnosis [7, 12, 15], and patients privately insured or insured by Medicare plus supplemental plans had lower likelihood of late-stage cancer diagnosis than persons with other types of insurance [33]. In these studies, race or ethnicity was classified into three groups: white, African-American, and all others. More detailed breakouts of races or ethnicities are needed for a better understanding of disparities, which is one major contribution of the research described here.

In addition to more detailed information regarding various races and ethnicities, other disparities by age, by sex, and by geographic area are important and need further study. While Henley et al. [14]) used mapping of state-specific estimates over total populations to demonstrate substantial geographic variation in the rates of late-stage diagnosis, this work is descriptive and does not help explain the geographic variation. To describe geographic disparities by race or ethnicity, the American Cancer Society published two reports (2012, 2013) which used mapping of state-specific estimates to demonstrate that the death rates associated with CRC exhibit different geographic patterns across men and women, whites, and African-Americans and that the death rates associated with BC have varied geographic patterns across white and African-American women. The reports did not provide information on geographic patterns for other races or ethnicities, but did demonstrate that disparities vary along both demographic and geographic dimensions.

The disentangling of geographic and demographic disparities is important for policies designed to reduce disparities in health outcomes. There is increasing recognition that health disparities vary widely among states in the USA, such that the effects of geographic place may be difficult to disentangle from racial, ethnic, social, economic, or cultural determinants of health [9, 10, 20, 22, 23, 26, 32]. To see why this is a problem, consider that the apparent national disparities measured for one racial group relative to whites may reflect disadvantages and cultural differences in the geographic places where they are most heavily concentrated and thus reflect geographic rather than racial disparities. That is, most residents of those regions may be similarly disadvantaged, regardless of race. If this is true, examining more local areas (e.g., states vs national) for evidence of demographic disparities would be comparing groups within a more similar context, thus enhancing separation of the demographic from the geographic disparity. If demographic disparities then exist within this local context, one could conclude it is the race, not the place that is resulting in the disparity. National statistics on health disparities derived from pooled data across states mask the socio-ecological differences found within more local areas (e.g., states) and may confound the geographic with the demographic disparities.

The purpose of this paper is to provide evidence of state-specific disparities in late-stage cancer diagnosis outcomes, by race or ethnicity, sex, or age groups, using individual-level data for the residents of each state. This evidence regarding disparities should be valuable in cancer control planning activities led by the individual states. Achieving this purpose requires population-based cancer registry data pooled over time so that each state has a large enough number of observations to ensure statistical reliability of the estimates.

Accordingly, we examined all newly diagnosed cancer cases during 2004–2009 from the United States Cancer Statistics (USCS) database, which is a population-based surveillance system of cancer registries with data representing 96 % of the US population [27]. The database was developed by a joint effort by the Centers for Disease Control and Prevention (CDC) and the National Cancer Institute (NCI) to provide a single, pooled-state database of reconciled, comparable cancer information geocoded at the local level to facilitate cancer control planning and evaluation [34]. This comprehensive database is now available inside National Centers for Health Statistics (NCHS) and Census Research Data Centers (RDCs) to qualified researchers [8].

The outcome of interest in this paper is the likelihood of being diagnosed at a late stage, for either BC or CRC. Nationally, the rates of late-stage diagnosis are higher for CRC than for BC, but these rates do vary considerably across the states [14]. Nationally, disparities in late-stage diagnoses among minorities and whites are greater for BC than for CRC, and women are more likely than men to be diagnosed at a late stage of CRC [21], but it is not known whether these disparities vary among the states. Finally, disparities among younger (than age 65) and older persons diagnosed with late-stage cancer are large for both cancer types in national analysis [21], but again, it is not known whether these disparities vary among the states.

The main contributions of this paper are to examine disparities in multiple dimensions, using predictive multivariate modeling rather than descriptive statistics. Utilizing the very large USCS database enables the generation of state-specific findings that can be reliably used to guide policy makers in their efforts to control cancer and its burdens.

## Methods

Understanding health disparities in late-stage diagnosis of cancer requires modeling of the sources of these disparities in a geospatial context using an approach that explicates the social ecology of the health behaviors. Omitting the social ecological variables increases the risk that racial, ethnic, sex, or age coefficients will exhibit omitted variables bias. To ascertain the independent effect of demographic status on the late stage of cancer outcome, the social and health market contexts must be held constant statistically by including these other covariates in the model. In this paper, we use a comprehensive multilevel model specification similar to that used in previous studies noted above and employ the same empirical specification for all state-specific analyses to enhance comparability across states.

More specifically, we used multilevel models to examine associations with late-stage cancer diagnosis from predictors at person and county levels. We merged county-level community characteristics with person-level records from the USCS registry data system (Table 1). Table 1 describes the variables used in the state-specific modeling.

**Table 1:** Variable descriptions for predictors included in the 40 state regressions, with national statistics

Variable		BC population: *N* = 981,457		CRC population: *N* = 558,568	
		Mean	sdev	Mean	sdev
Outcome: whether cancer patient was diagnosed at a late stage (regional or distant =1, else = 0)		0.308	0.461	0.543	0.498
Person-level predictors (source: USCS database: CDC 2015)					
Female (reference male)	Binary indicator that person is female	1.000	0.000	0.487	0.500
White (reference)	Binary indicator that person is white	0.773	0.419	0.765	0.424
African-American	Binary indicator that person is African-American	0.101	0.301	0.112	0.315
Hispanic	Binary indicator that person is Hispanic	0.081	0.273	0.080	0.271
Asian/Pacific Islander	Binary indicator that person is Asian/PI	0.033	0.177	0.031	0.172
Race all other	Binary indicator that person is other	0.013	0.090	0.013	0.088
Age less than 40	Binary indicator that person is <40	0.051	0.220	0.027	0.163
Age 40–49	Binary indicator that person is 40–49	0.193	0.395	0.083	0.275
Age 50 to 64	Binary indicator that person is 50–64	0.379	0.485	0.314	0.464
Age 65 to 74	Binary indicator that person is 65–74	0.201	0.400	0.250	0.433
Age 75 plus (reference)	Binary indicator that person is 75+	0.176	0.381	0.325	0.468
County-level predictors for counties of residence (source: RTI 2015)					
Isolation black	Higher isolation index represents a lower chance that minorities reside among whites, with a value of 1 indicating a perfectly segregated society, 2005	0.257	0.214	0.259	0.217
Isolation Asian		0.072	0.086	0.068	0.085
Isolation Hispanic		0.216	0.203	0.209	0.205
Managed care	% Population insured by managed care plans, 2001	15.9	14.7	15.3	14.7
Distance (miles)	Based on 100 % FFS Medicare utilization of mammography or endoscopy services, 2006	6.02	6.10	5.15	4.80
Screening rate (%)		23.60	3.18	11.05	1.43
Percent uninsured (%)	% Population < age 65 with no health insurance, 2005	17.73	5.45	17.75	5.49

We specify a two-level random intercept logit model for the late-stage diagnosis with patients nested in counties. We used a multilevel modeling (MLM) framework for estimation because we wanted to fit the regression to individuals while accounting for systematic, unexplained variation among counties. Ignoring these county-level effects, when they are important, is tantamount to having omitted variables in the model, which can bias individual-level coefficient estimates, such as the disparities estimates [11]. Because we seek unbiased estimates of disparity coefficients for each state, we adopted the two-level random intercept logistic regression estimated here. We estimated the regressions using SAS GLIMMIX (SAS/STAT 2016). We estimated the logistic multilevel regression model of the binary cancer stage outcome separately for each cancer type and each state. The models for each cancer type used identical predictors, except CRC included an indicator to differentiate males and females.

### Study Population and Data

We obtained all BC and CRC cases diagnosed in 2004–2009 from the USCS database, available to researchers with approved projects inside the NCHS and Census RDCs. In this study, we required information regarding the county of residence for each cancer patient so that we could include community contextual factors (uninsured, area screening, managed care penetration, screening access/availability, and residential isolation by race or ethnicity) in our multilevel modeling. While all but three states (Kansas, Maryland, Minnesota) participate in the USCS registry data system, four do not allow use of county of residence information (Illinois, Michigan, Missouri, Ohio). We excluded from the analysis these seven states and an additional state, Virginia, because registry data were not available until 2007. We also excluded Alaska and Hawaii, because their county designations are much different than for mainland states (each island is a county in Hawaii, and Alaska has boroughs rather than counties), and data are missing for some county-level constructs.

After exclusions, this resulted in 40 states over the 2004–2009 period. We restricted the sample to adults of all ages with a first cancer diagnosis in 2004–2009. We excluded records when BC or CRC were not the primary cancers, records with unknown cancer stage or unstaged cancer, or when diagnosis was by autopsy (<1 % of all cases). For BC, we excluded males. These restrictions yielded a CRC study population of 558,568 individuals and a BC study population of 981,457 individuals. The state with the smallest number of cases was Wyoming, with 1916 BC cases and 1163 CRC cases. The state with the largest number of cases was California, with 135,895 BC cases and 71,458 CRC cases. Thus, the cancer population sizes are sufficient in each state to ensure the statistical power needed to estimate the various demographic disparities (Table 2).

**Table 2:** Population counts and late-stage diagnosis rates, by state

State abbreviation	BC cases	Proportion BC cases diagnosed at late stage	CRC cases	Proportion CRC cases diagnosed at late stage
AL	20,788	0.35	13,660	0.52
AZ	20,517	0.31	10,520	0.55
AR	11,501	0.34	7556	0.59
CA	135,895	0.31	71,458	0.56
CO	18,275	0.30	8810	0.53
CT	17,949	0.26	9213	0.50
DE	3982	0.28	2195	0.55
FL	79,402	0.30	46,239	0.55
GA	35,706	0.32	19,681	0.55
ID	5160	0.32	2730	0.60
IN	25,045	0.30	16,100	0.52
IA	12,926	0.29	8412	0.58
KY	17,605	0.31	12,307	0.53
LA	17,390	0.34	11,754	0.57
ME	6820	0.27	3944	0.50
MA	33,513	0.25	17,264	0.50
MS	11,236	0.36	8002	0.55
MT	4163	0.29	2323	0.57
NE	7395	0.30	4689	0.58
NV	8366	0.32	4950	0.59
NH	6274	0.26	3354	0.49
NJ	40,729	0.31	22,519	0.55
NM	6824	0.31	3922	0.56
NY	89,085	0.29	49,076	0.53
NC	38,335	0.31	21,620	0.53
ND	2699	0.32	1885	0.52
OK	14,844	0.34	8941	0.55
OR	16,810	0.29	8200	0.60
PA	59,408	0.31	38,692	0.52
RI	5160	0.28	2838	0.51
SC	19,641	0.32	11,141	0.54
SD	3356	0.30	2161	0.51
TN	25,601	0.33	15,536	0.53
TX	79,097	0.34	44,674	0.56
UT	6935	0.34	3589	0.51
VT	3329	0.24	1618	0.50
WA	27,921	0.29	12,792	0.59
WV	7936	0.31	5951	0.51
WI	23,791	0.29	12,236	0.52
WY	1916	0.32	1163	0.54
Total	981,457	0.31	558,568	0.54

We defined whites as the reference group in the race or ethnicity assessments and included African-American, Asian, Hispanic, and “all other” as minority group designations. We include age groups <40, 40–49, 50–64, 65–74, and 75. Thus, our sample includes all adults with BC and CRC, in contrast to Henley et al. [14]) who truncated the population to include only those individuals with the age of 50+. Population data are from the USCS database [8], and county-level data describing contextual characteristics of communities were obtained from the RTI Spatial Impact Factor Database (https://rtispatialdata.rti.org), which derives from numerous public sources, such as the Centers for Medicare and Medicaid Services and the US Census Bureau.

To the extent that states act independently to promote their cancer control efforts, it is reasonable to model each state as a separate system. This approach allows us to examine unique effects of disparity covariates (e.g., race or ethnicity relative to whites) on the outcome variables (i.e., likelihood of late-stage diagnosis) and compare results across states. Independent modeling of each state’s population in multilevel models, which include person-level and community-level covariates, results in robust disparity estimates that are adjusted to reflect local market and socio-economic factors. Thus, we can determine whether minorities and whites have different outcomes, when facing the same socio-ecological contexts.

### Statistical Analysis

We estimated state-level disparities using multilevel logistic models for individuals within each state, including as covariates the same personal demographic (level 1) and community (level 2) constructs in each state model, analyzed separately for BC and CRC. Using the separate regression results for each of the 40 states and two cancer types, we examined the effects of race-ethnicity, gender (for CRC), and age on the likelihood of late-stage diagnosis for BC and CRC. Specifically, we wanted to answer the following research questions:

- Are African-Americans, Asians, Hispanics, or Native Americans more likely to be diagnosed at a late stage for BC or CRC than whites, and if so, in which states?
- Are women more likely to be diagnosed at a late stage for CRC than men, and if so, in which states?
- Are older persons more likely to be diagnosed at a late stage for BC or CRC than younger persons, and if so, in which states?

### Translation of Findings

To display the results of model estimates for the state-specific analysis would require 40 tables, one for each state included in the database, to display separately estimated models of late-stage diagnosis of BC and CRC. As an alternative, the *statistically significant* (*p* value ≤0.05) estimates of disparities from these state-specific models were translated to the reader via maps. We displayed the significant disparity coefficients for all 40 states together, using a set of maps, one for each racial, ethnic, or age-related disparity estimate. Using this method, each state’s estimate was independent of estimates in other states, reflecting the very different social, political, economic, regulatory, and cultural environments across states.

Each disparity estimate was classified into three categories to show if the disparity was significantly higher than the reference group (positive, associated with significantly higher likelihood of late-stage cancer diagnosis), significantly lower than the reference group (negative, associated with significantly lower likelihood of late-stage cancer diagnosis), or not statistically different (zero, no significant difference in the likelihood of late-stage cancer diagnosis from the reference group). If we had used exponentiation to convert the model estimates into odds ratios, the corresponding groupings would be as follows: significantly greater than 1 (associated with significantly higher odds of late-stage cancer diagnosis than the reference group), not significantly different from 1 (not significantly different from reference group), or significantly lower than 1 (associated with significantly lower odds of late-stage cancer diagnosis than the reference group).

## Results

Sample statistics are presented in Table 1, for the pooled national data. About 10–11 % of the cancer population are African-American, and about 3 % are Asian, 8 % are Hispanic, and ∼1 % are other races combined; the vast majority are white, the reference group. Almost half (48.7 %) of CRC cases are females. More than 62 % of the BC sample are younger than 65 years old, whereas less than 43 % of the CRC sample are people younger than 65. Thus in general, BC seems to be a “younger” disease at onset than CRC. In the USA, screening protocols reflect these disease trajectories. The BC screening protocols recommended screening to begin at age 40, while CRC screening guidelines recommended screening to begin at age 50.

**Fig. 1:**
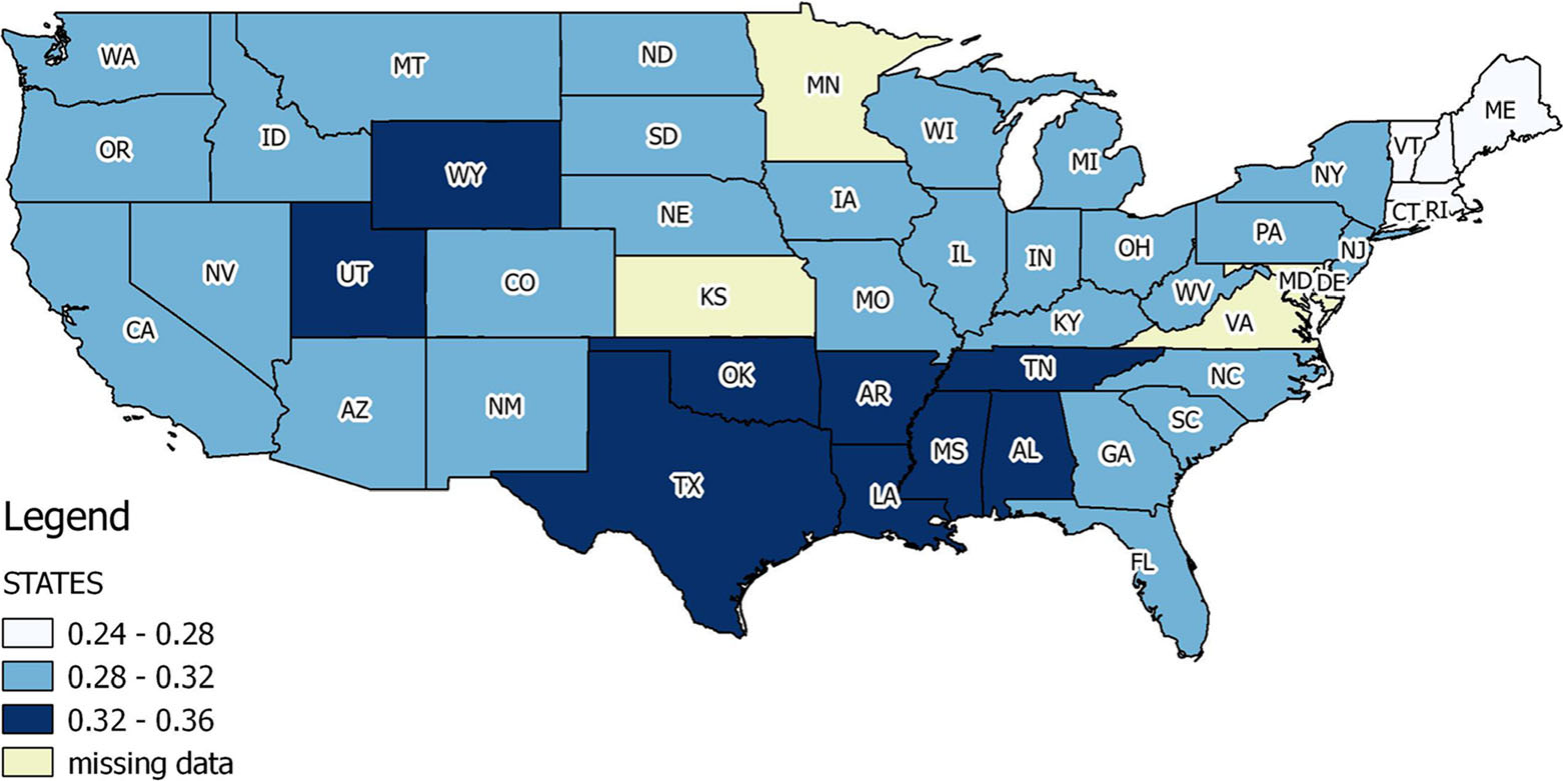
State-specific proportions of breast cancer cases diagnosed at a late stage, 2004–2009

**Fig. 2:**
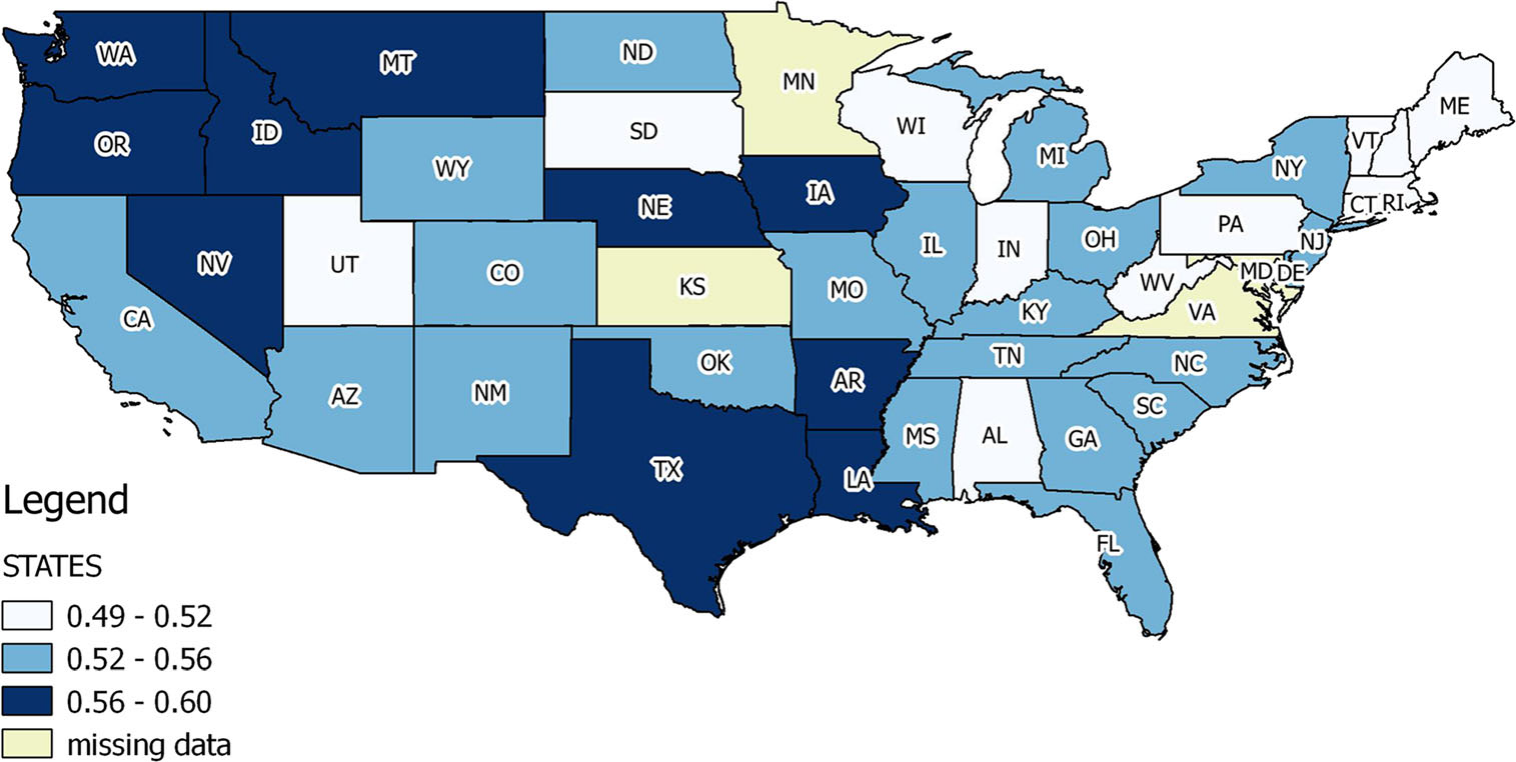
State-specific proportions of colorectal cancer cases diagnosed at a late stage, 2004–2009

Over all individuals (Table 1), the national average of late-stage BC cases is 30.8 % and the national average of late-stage CRC cases is 54.3 %. We use mapping to show state-specific overall late-stage cancer diagnosis rates for BC (Fig. 1) and CRC (Fig. 2). Vermont has the lowest percentage of late-stage BC diagnoses (24.3 %), and Mississippi has the highest (36.4 %), with the mean represented by the state of New Jersey (30.8 %). For CRC, the state with the lowest percentage of late-stage diagnoses is New Hampshire (48.5 %), while the highest is Oregon (60 %) and the mean is represented by Arizona (54.3 %).

**Fig. 3:**
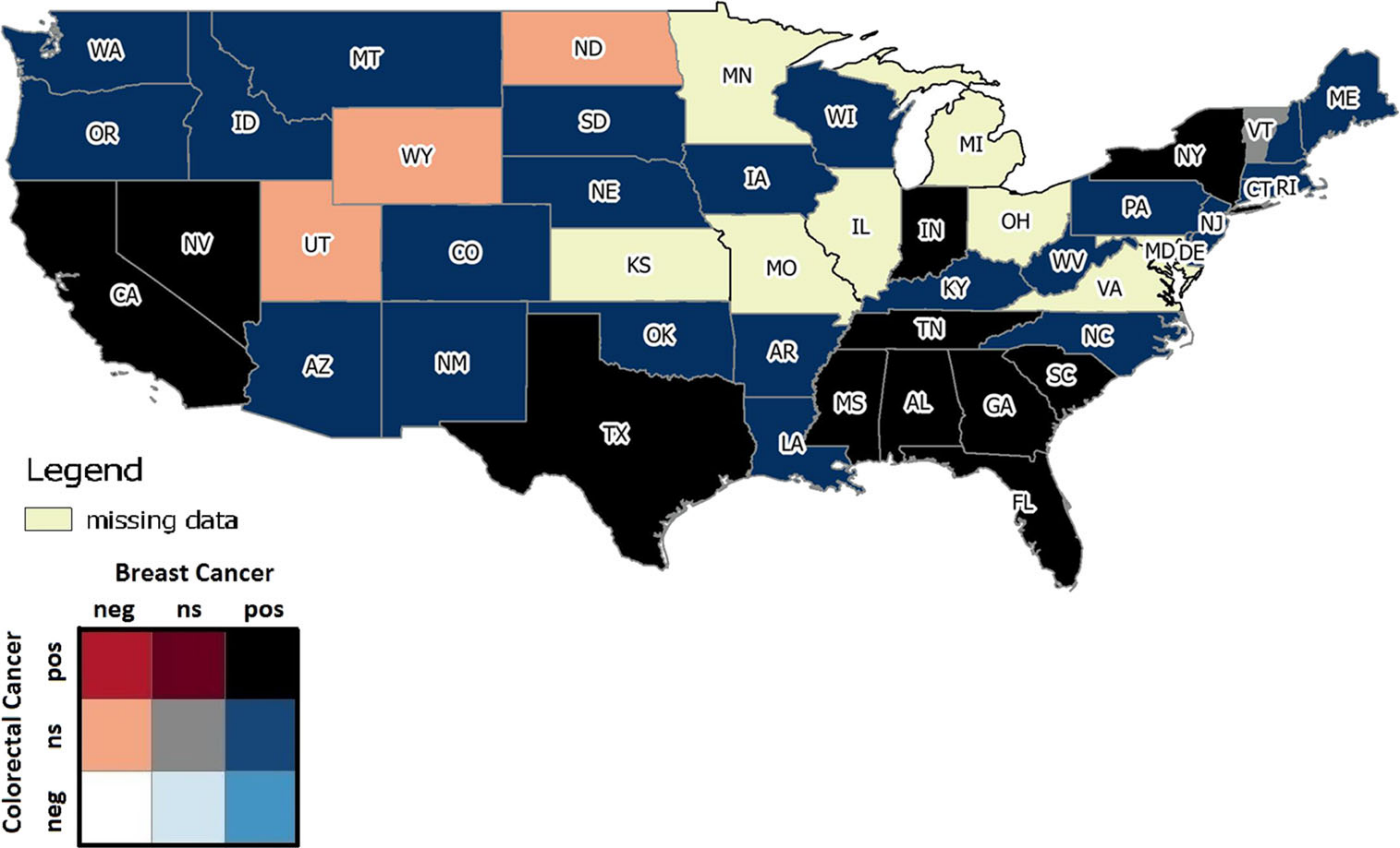
State-specific disparities in late-stage diagnosis of BC or CRC, by African-Americans versus whites

**Fig. 4:**
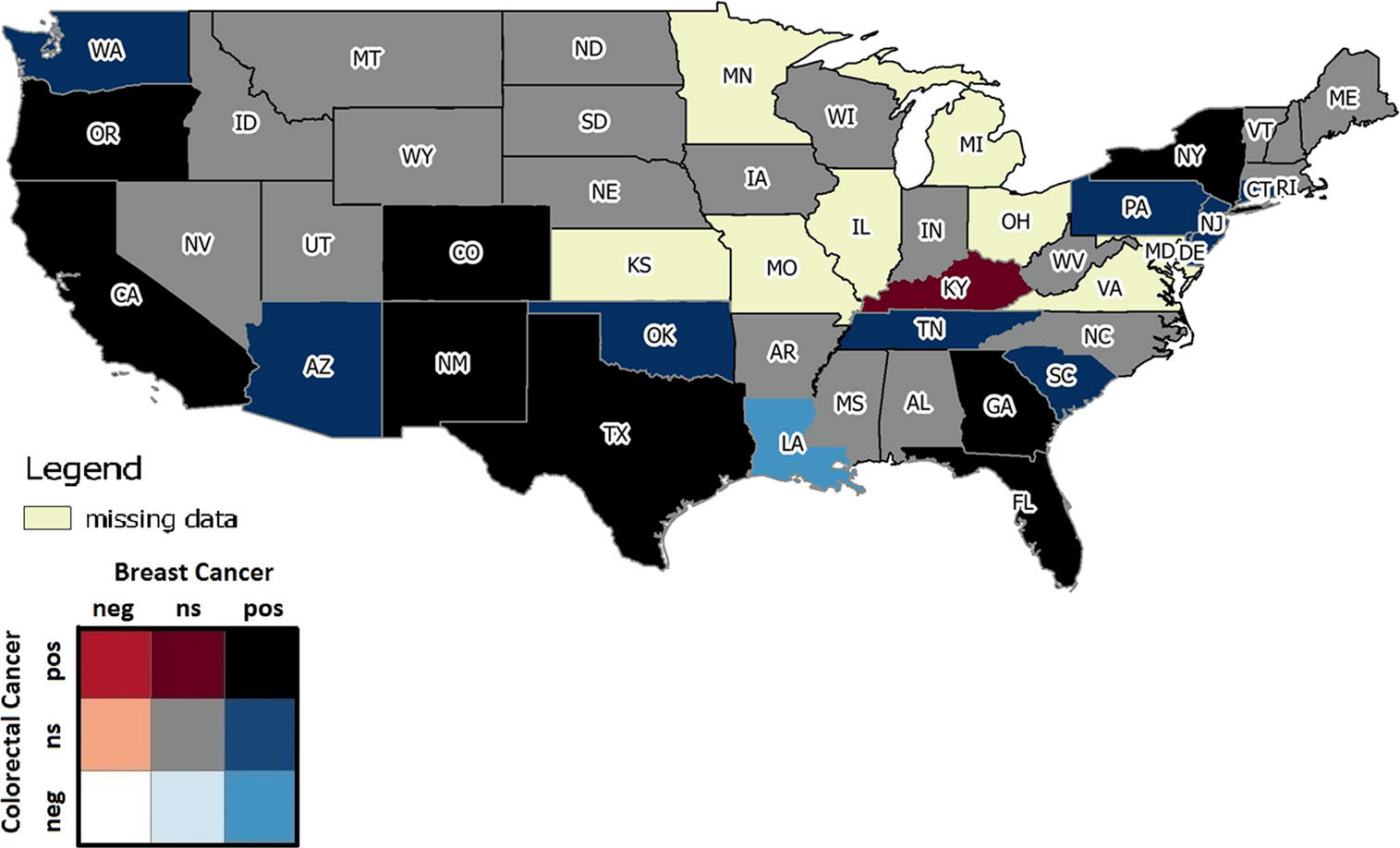
State-specific disparities in late-stage diagnosis of BC or CRC, by Hispanics versus whites

**Fig. 5:**
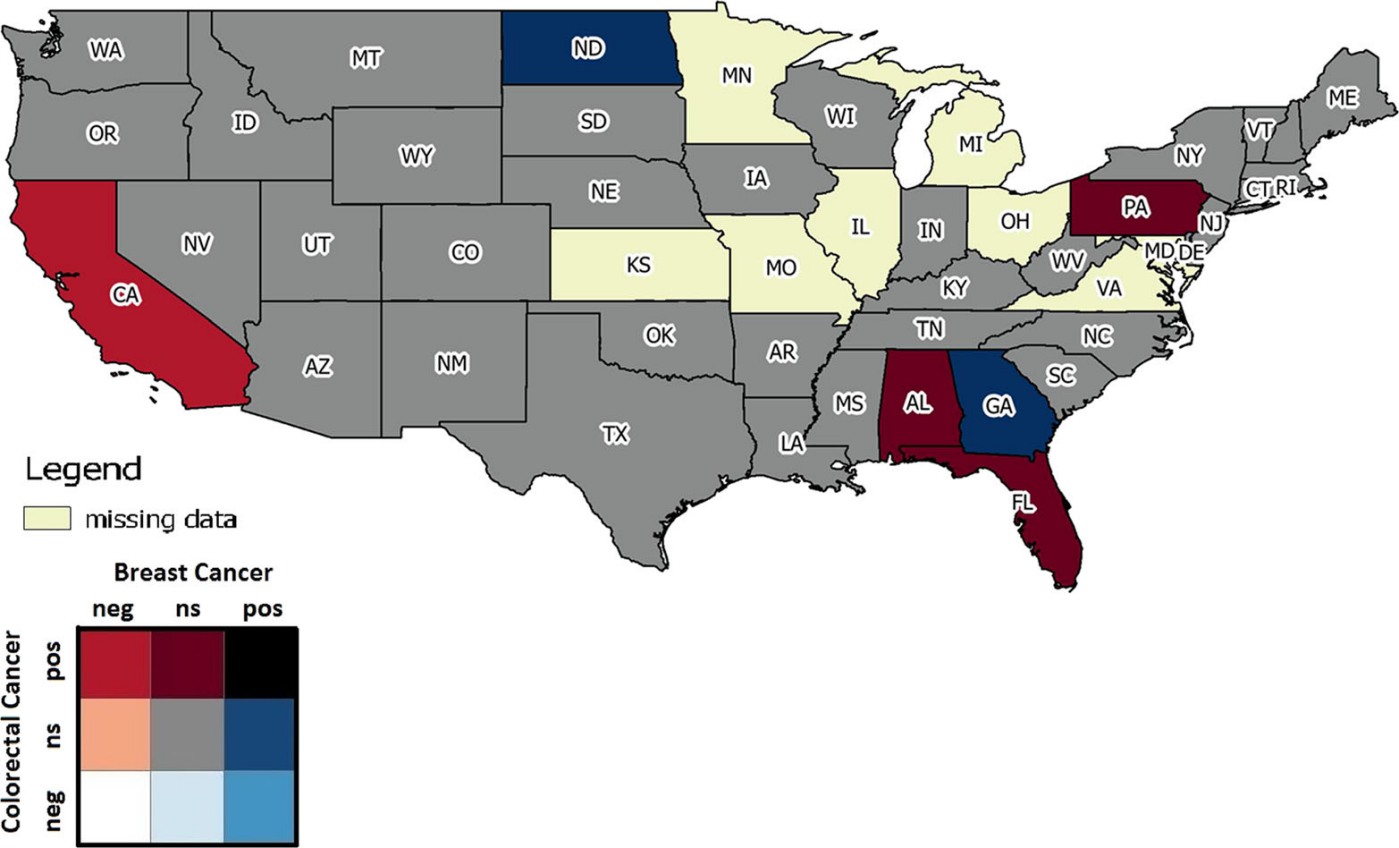
State-specific disparities in late-stage diagnosis of BC or CRC, by Asians versus whites

**Fig. 6:**
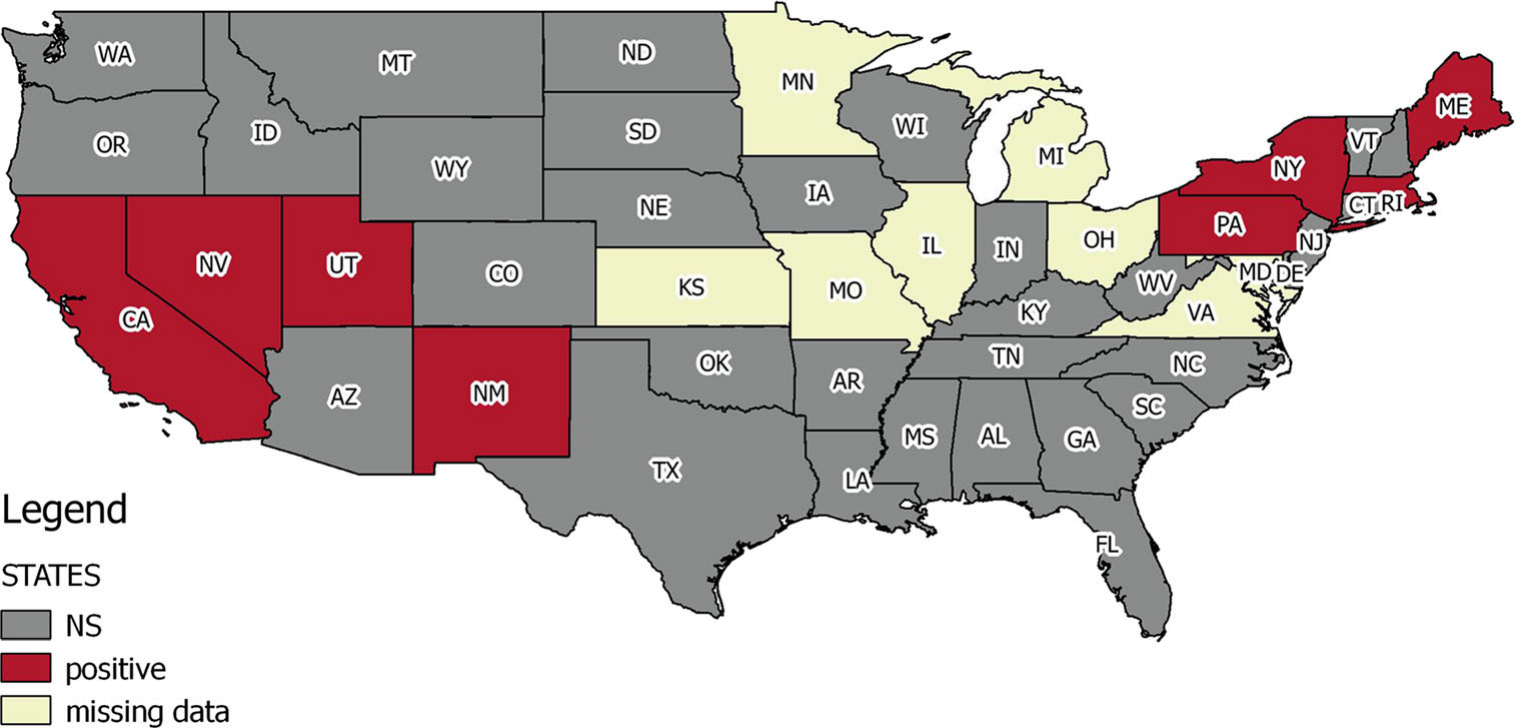
State-specific disparities in late-stage diagnosis of CRC, by women versus men

**Fig. 7:**
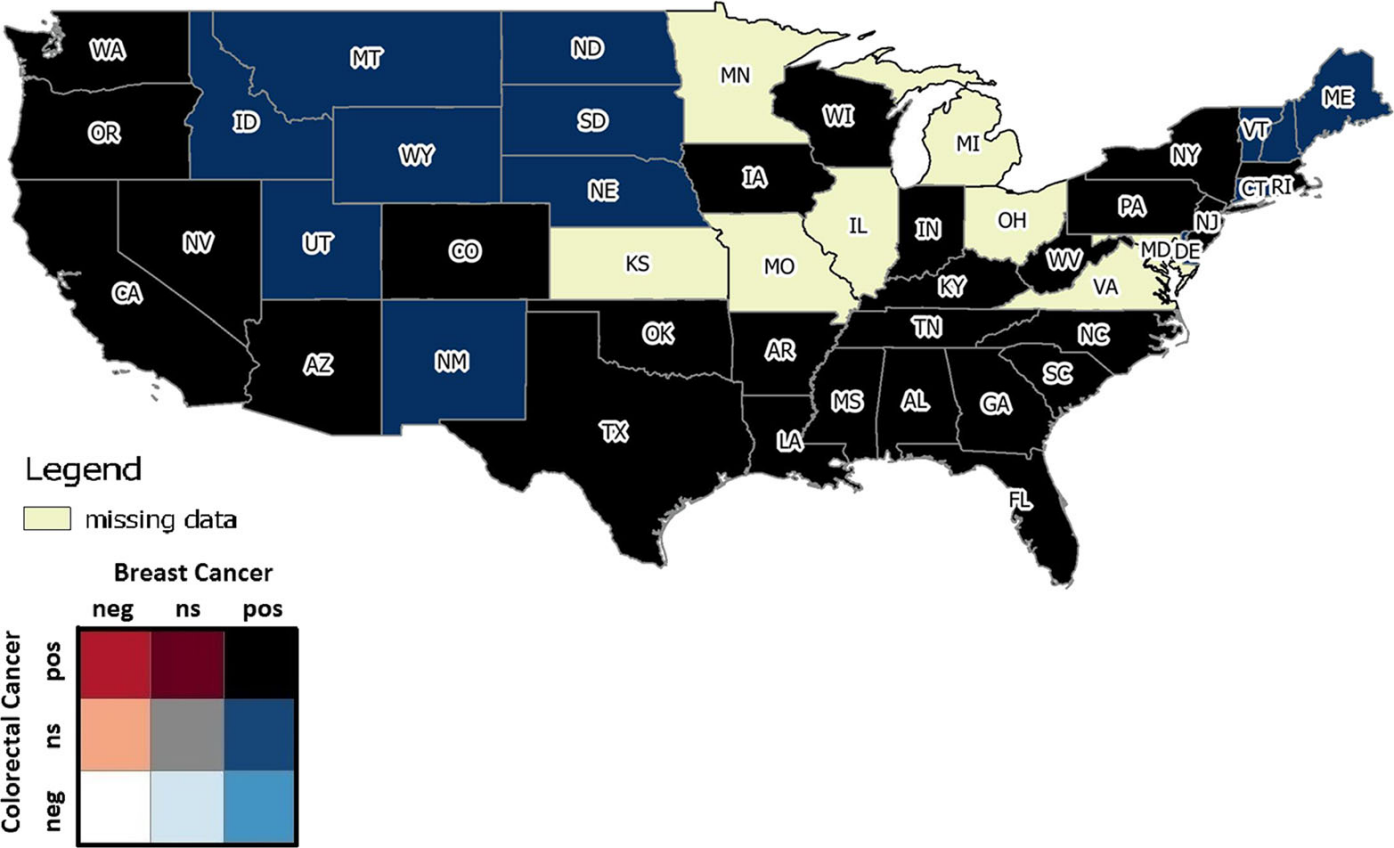
State-specific disparities in late-stage diagnosis of BC or CRC, by group aged <40 versus 75+

**Fig. 8:**
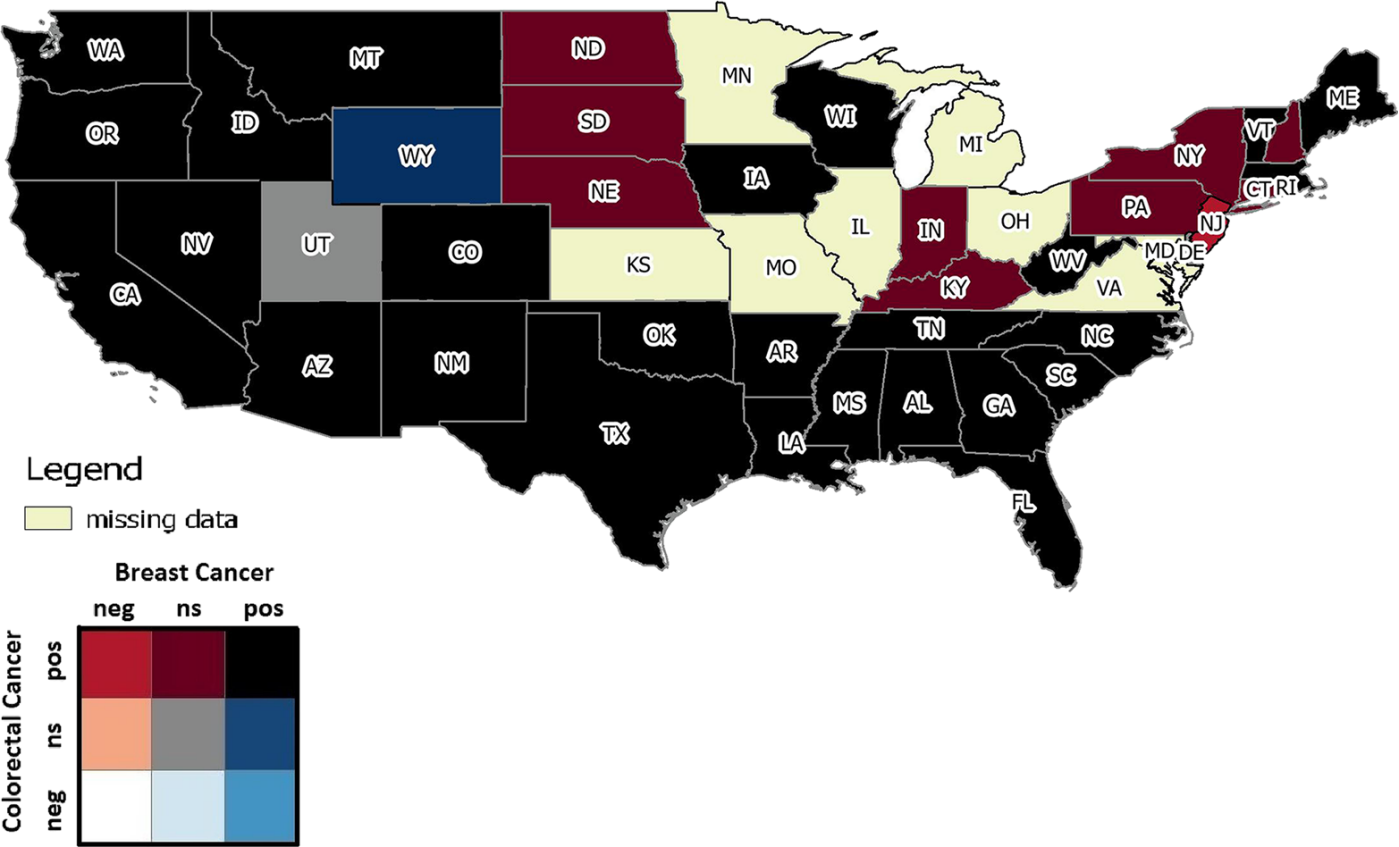
State-specific disparities in late-stage diagnosis of BC or CRC, by group aged 40–49 versus 75+

**Fig. 9:**
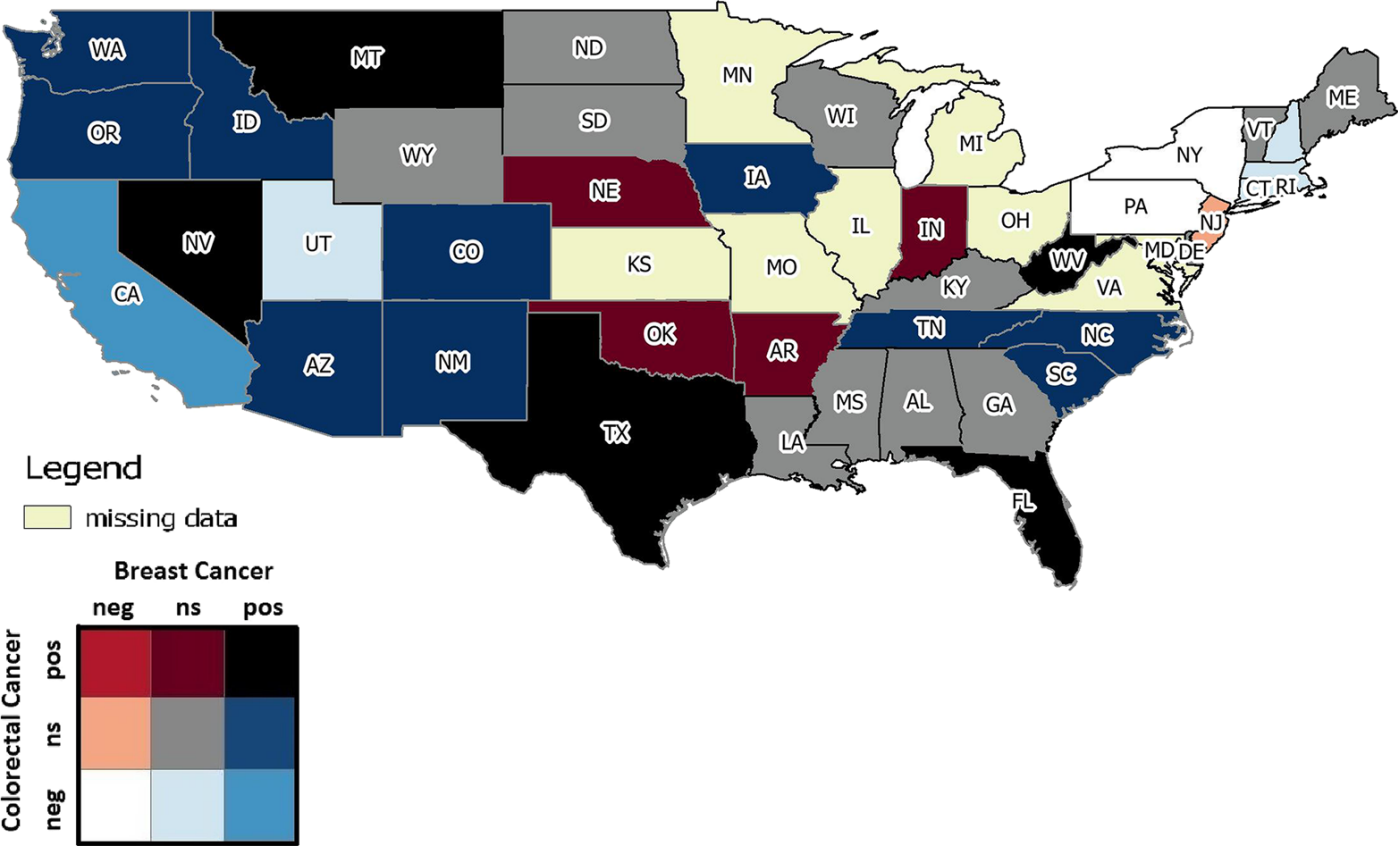
State-specific disparities in late-stage diagnosis of BC or CRC, by group aged 50–64 versus 75+

**Fig. 10:**
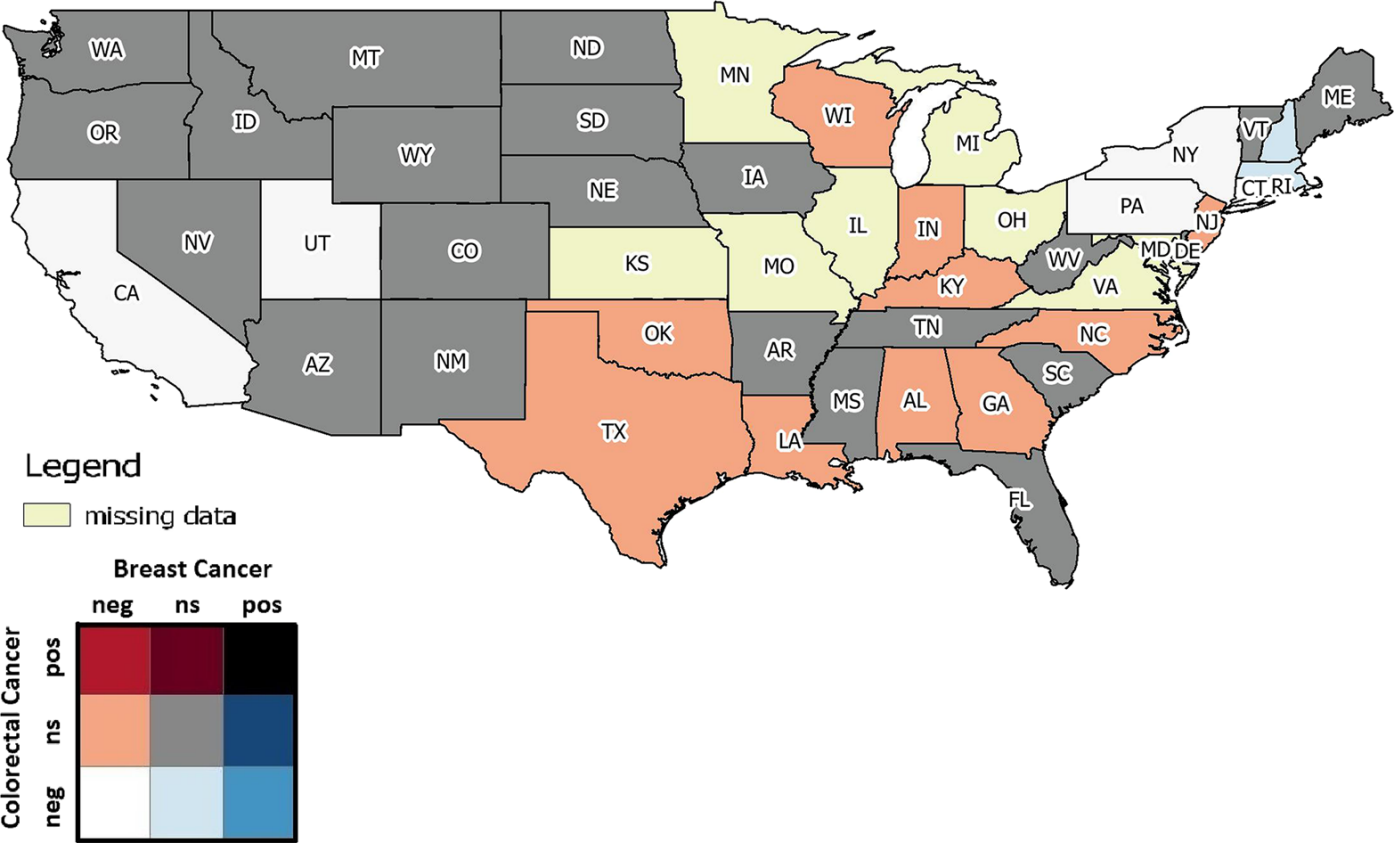
State-specific disparities in late-stage diagnosis of BC or CRC, by group aged 65–74 versus 75+

The state-specific disparities effect estimates are presented in Figs. 3, 4, 5, 6, 7, 8, 9, and 10. In these maps, we coded an estimate “0” and used gray coloring in the maps whenever there was no statistically significant disparity estimate in that state’s regression (using a *p* value >0.05), for each demographic factor. Positive disparity estimates with a statistically significant *p* value (*p* value ≤0.05) reflect higher likelihood than the reference group of a late-stage diagnosis, while negative effect estimates with a statistically significant *p* value reflect a “reverse” disparity (i.e., lower likelihood than the reference group). Only statistically significant estimates are colored with shades other than gray in the maps.

For the research question concerning effect of race or ethnicity on late-stage BC and CRC, we found that African-Americans (Fig. 3), Hispanics (Fig. 4), and Asians (Fig. 5) are more likely to be diagnosed at a late stage for BC or CRC or both than whites in many states. However, there is a reverse disparity (e.g., designated group less likely than reference group to be diagnosed at a late stage)—where African-Americans are less likely than whites for late-stage BC diagnosis—in Utah, Wyoming, and North Dakota. For Asians, there is a reverse disparity for BC in California, but an ordinary disparity (higher likelihood) for CRC. For Hispanics, there is a reverse disparity for CRC in Louisiana, but an ordinary disparity (higher likelihood) for BC.

For gender effects on late-stage diagnosis of CRC, Fig. 6 shows that eight states colored in red (CA, NV, NM, UT, PA, NY, MA, ME) had significantly higher likelihood that women would be diagnosed at a late stage for CRC, as compared to men. (Note that MA is located above CT and RI.)

Figures 7, 8, 9, and 10 show age effects on late stage for BC and CRC, in which each younger age group was compared to the age 75 and older group. It is apparent that the age 65–74 group (Fig. 10) is less likely than the oldest group to be diagnosed late, in 18 states (colored white, light blue, and pink). However, the two groups under age 65 are more likely than the 75+ group to be diagnosed late, in the majority of states (Figs. 7 and 8). The youngest group under age 40 (Fig. 7) is more likely to be diagnosed late for BC or both cancer types than those aged 75+, in every state. For the group aged 40–49 (Fig. 8), all but two states show significant disparities, but NJ shows a reverse disparity for BC coupled with an ordinary disparity for CRC (bright red) and Utah shows no significant disparity. The group aged 50–64 (Fig. 9) shows a lot of variability across the USA. Five states have a significant disparity in both cancer types relative to the oldest group (FL, WV, TX, MT, NV), four show a significant CRC disparity (IN, AR, NE, OK), and eleven others show a significant disparity for BC (WA, OR, ID, CO, AZ, NM, IA, TN, NC, SC, CA). The remaining 20 states show negative (colors pink, light blue, or white) or no disparities (gray) relative to the oldest group. The age 65–74 group (Fig. 10) shows reverse disparities across about half of the states (pink, light blue, white) with the remaining states showing no disparity.

In addition, compared to people aged 75+, Fig. 9 also shows a reverse disparity for CRC (light blue) for people aged 50–64 in states of Utah, New Hampshire, Massachusetts, and Connecticut; a reverse disparity for BC (pink) in New Jersey; and reverse disparities for both cancer types in Pennsylvania and New York. Similarly, Fig. 10 shows an abundance of states with reverse disparities for BC (pink) or CRC (light blue) or both (white) for people aged 65–74 compared to those aged 75+.

## Discussion and Summary

Using the USCS database for new breast or colorectal cancers diagnosed during 2004–2009, in 40 of the United States, we identified similar raw proportions of late-stage diagnosis of cancers to those reported by Henley et al. [14]). Our study filled important gaps by estimating detailed demographic disparities for each state, identified as effect estimates from multivariate and multilevel models which control for omitted variables bias and yield reliable effect estimates. Findings demonstrated considerable variability across states in the demographic disparity dimensions we examined, which included race or ethnicity, age, and gender. Thus, the national statistics from previous studies have been parsed into their sub-national components which may be helpful in forming state policy to improve cancer control efforts.

For example, national statistics suggest that African-Americans are more likely to be diagnosed at a late stage for both BC and CRC as compared to whites (e.g., [14, 21]). This is apparently true in many states (Fig. 3, black coloring), and the disparity is actually greater for BC than for CRC (Fig. 3, black and dark blue coloring). The national statistics do not pertain in all states, however. There are a few states where a reverse disparity seems to exist for African-American women (Fig. 3, pink coloring). There are also a few states in New England with no apparent disparities for African-Americans for either type of cancer (gray coloring). Finally, national statistics suggest that the African-American disparity is stronger for BC than for CRC, which is borne out in mapping the individual state disparities (Fig. 3) which reveals the local patterns across the states.

National statistics also suggest that breast cancer patients under age 40 are generally at higher risk for more aggressive tumors that are more difficult to treat and may pose worse outcomes [28]. A particular subtype of BC, inflammatory BC, is always diagnosed at a late stage and is an example of extremely aggressive BC inflicting younger women, especially African-Americans [13]. More aggressive tumors increase the odds of being diagnosed for BC at later stages, so our finding that women in the youngest age group (under age 40, Fig. 7) are more likely to be diagnosed late for BC in every state resonates with the clinical data regarding more aggressive tumors among younger women. Our findings generally demonstrate significant disparities in age, gender, and race or ethnicity for late-stage BC and CRC. However, disparities are not similar across the states for the two cancer types. Equal findings across the two cancer types would result in maps with only white, gray, and black colors. The states colored bright red, or bright blue indicate situations where disparities for one cancer type are positive (higher than the reference group), while disparities for the other type are negative (lower than the reference group). In these rare situations, disparity findings are directly opposite across the cancer types. Four cases of these opposites are found. First, in the Hispanic disparities map (Fig. 4), this situation exists in Louisiana, where Hispanics have significantly higher likelihood of late-stage BC diagnosis, but significantly lower likelihood of late-stage CRC diagnosis than whites. Second, in the Asian disparities map (Fig. 5), this situation exists in California, where Asians have significantly higher likelihood of late-stage CRC diagnosis, but significantly lower likelihood of late-stage BC diagnosis than whites. Third, in the aged 40–49 disparities map (Fig. 8), this situation exists in New Jersey, where cancer patients aged 40–49 have significantly higher likelihood of late-stage CRC diagnosis, but significantly lower likelihood of late-stage BC diagnosis than the group aged 75+. Fourth, in the aged 50–64 disparities map (Fig. 9), this situation exists in California, where cancer patients aged 50–64 have significantly higher likelihood of late-stage BC diagnosis, but significantly lower likelihood of late-stage CRC diagnosis than the group aged 75+.

National statistics suggest overall trends, but mask important differences that may exist from state to state. When population health data are available to researchers, the variability across the landscape can be reliably explored, using population sizes sufficient to yield statistically significant findings at sub-national scales. A limitation is that only 40 of the 50 United States provided the full dataset needed for this analysis. Perhaps the findings from the states included will encourage more reluctant states to join the USCS database effort.

This study has established disparities benchmarks based on the 2004–2009 period for the late-stage diagnosis of BC and CRC. Further research inside the RDC labs can assess these disparities in the more current time, beginning in 2010, when landmark health policy legislation was enacted in the USA. The Patient Protection and Affordable Care Act of 2010 [1] mandated that all new private insurance plans cover all costs of colonoscopy for CRC screening and all costs of mammography for BC screening, among other preventive services. Prior to the act, copayments for colonoscopies had included a significant percentage of procedure costs as out of pocket expenditures and were subject to annual deductibles, and this pertained to elderly persons on Medicare as well as younger persons [25]. Another change that was implemented with the ACA in 2010 was the ban on insurers’ use of pre-existing condition clauses to refuse to underwrite or charge unaffordable high premiums to the sick.

After 2010, we expect that a surge of unmet, pent-up demand among the sick for preventive services would result in lower late-stage diagnosis rates in the future. Time will tell whether our expectation is met, and the USCS database in the RDC will be an excellent data source for testing hypotheses such as these. The value of this database for disparities research in cancer control has barely been tapped and will generate a lot of valuable research in the future.
